# Automatic segmentation of mandibular canal using transformer based neural networks

**DOI:** 10.3389/fbioe.2023.1302524

**Published:** 2023-11-17

**Authors:** Jinxuan Lv, Lang Zhang, Jiajie Xu, Wang Li, Gen Li, Hengyu Zhou

**Affiliations:** School of Pharmacy and Bioengineering, Chongqing University of Technology, Chongqing, China

**Keywords:** mandibular canal, transformer, feature fusion, segmentation, CBCT

## Abstract

Accurate 3D localization of the mandibular canal is crucial for the success of digitally-assisted dental surgeries. Damage to the mandibular canal may result in severe consequences for the patient, including acute pain, numbness, or even facial paralysis. As such, the development of a fast, stable, and highly precise method for mandibular canal segmentation is paramount for enhancing the success rate of dental surgical procedures. Nonetheless, the task of mandibular canal segmentation is fraught with challenges, including a severe imbalance between positive and negative samples and indistinct boundaries, which often compromise the completeness of existing segmentation methods. To surmount these challenges, we propose an innovative, fully automated segmentation approach for the mandibular canal. Our methodology employs a Transformer architecture in conjunction with cl-Dice loss to ensure that the model concentrates on the connectivity of the mandibular canal. Additionally, we introduce a pixel-level feature fusion technique to bolster the model’s sensitivity to fine-grained details of the canal structure. To tackle the issue of sample imbalance and vague boundaries, we implement a strategy founded on mandibular foramen localization to isolate the maximally connected domain of the mandibular canal. Furthermore, a contrast enhancement technique is employed for pre-processing the raw data. We also adopt a Deep Label Fusion strategy for pre-training on synthetic datasets, which substantially elevates the model’s performance. Empirical evaluations on a publicly accessible mandibular canal dataset reveal superior performance metrics: a Dice score of 0.844, click score of 0.961, IoU of 0.731, and HD95 of 2.947 mm. These results not only validate the efficacy of our approach but also establish its state-of-the-art performance on the public mandibular canal dataset.

## 1 Introduction

The mandibular canal (MC) is a tubular anatomical structure situated within the mandible and chiefly houses the inferior alveolar nerve (IAN) and associated vasculature (Agbaje et al., 2017). This nerve shares a critical relationship with the third molar (Rai et al., 2014). Any insult to the MC can lead to adverse outcomes such as patient discomfort, acute pain, or even facial paralysis (Al-Juboori et al., 2014). Therefore, precise segmentation of the MC from imaging modalities is instrumental for clinicians to appreciate the spatial relationship between the MC and adjacent anatomical landmarks, thereby minimizing the risk of iatrogenic nerve injury during surgical interventions (Li et al., 2022). Owing to the cumbersome and error-prone nature of manual delineation, automated segmentation of the MC from radiological images has emerged as a focal point in dental research (Usman et al., 2022).

With the advent of advanced deep learning techniques, neural network-based segmentation of oral structures has shown significant progress (Cui et al., 2022; Fontenele et al., 2023). However, the segmentation of the mandibular canal still falls short when compared to other anatomical structures. The primary challenges are multifaceted. First, the mandibular canal occupies a minute fraction of the overall CBCT image, which can lead the neural network to prioritize the background over the target foreground. Second, the low contrast of CBCT images makes it difficult to distinguish the mandibular canal from surrounding tissues, often resulting in blurred or indistinct boundaries. Traditional segmentation approaches such as region growing, level set, thresholding, and model matching have proven insufficient for overcoming these obstacles (Kainmueller et al., 2009; Abdolali et al., 2017). U-Net-based architectures have exhibited excellent performance across various domains since their introduction. Nonetheless, they often lack the capability to provide holistic information, causing them to neglect the topological structure of the mandibular canal during segmentation tasks (Jaskari et al., 2020; Lahoud et al., 2022). In recent years, Transformer-based encoder-decoder frameworks [e.g., TransUNet (Chen et al., 2021), UNETR (Hatamizadeh et al., 2022a), UNETR++ (Shaker et al., 2022)] have emerged, demonstrating promising results (Li et al., 2023). These Transformer-based methodologies utilize a global mechanism to capture features over long distances, addressing the limitations of CNN-based networks. However, the existing Transformer-based segmentation methods predominantly focus on larger organs, and they still do not provide effective solutions for segmenting the mandibular canal, which has smaller voxel sizes.

To address the aforementioned challenges, we have enhanced the Swin-UNetR model specifically for mandibular canal segmentation. We also incorporate a pixel-level feature fusion module to augment the model’s capability to discern finer details of the mandibular canal. To mitigate the severe class imbalance between the foreground and background, as well as the low contrast prevalent in mandibular canal data, we introduce a cropping technique grounded in mandibular foramen localization and a contrast enhancement strategy based on Contrast-Limited Adaptive Histogram Equalization (CLAHE). Given the topological continuity of the mandibular canal, we employ clDice as the model’s loss function. Moreover, to improve model robustness, we propose a straightforward yet effective deep label fusion technique that capitalizes on the sparse data in the dataset. Our main contributions can be summarized as follows:

(1) We introduce an enhanced Transformer-based segmentation network tailored for mandibular canal segmentation, offering a novel avenue for accurate segmentation of this complex structure.
(2) We proposed a pixel-level feature fusion module to improve the model’s detail perception ability, and improve the model’s segmentation accuracy and convergence speed.
(3) We introduce a cropping method that autonomously localizes the mandibular and mental foramina, coupled with an image contrast enhancement strategy, as preprocessing steps to address the challenges of category imbalance and unclear mandibular canal boundaries. Furthermore, our depth expansion technique is used to generate fused label datasets, enhancing the model’s robustness.

The remainder of the paper is structured as follows: Section 2 reviews related work in mandibular canal segmentation. Section 3 provides a comprehensive description of our proposed method. Section 4 discusses the materials and implementation details. In Section 5, we present the results along with comparative analyses. Section 6 contains the analysis and discussion of our work. Finally, Section 7 concludes the paper.

## 2 Related work

In the fisrt chapter, we delineated the broader research context, current state of the field, and specifically emphasized the importance and challenges associated with mandibular canal segmentation. In the subsequent chapters, we will delve deeper into the historical development of various mandibular canal segmentation techniques. These methods can be broadly categorized based on the underlying technology into traditional image processing techniques, CNN (Convolutional Neural Network)-based approaches, and Transformer-based segmentation methods.

### 2.1 Traditional image processing-based segmentation method

To address the clinical issue of solely relying on manual segmentation of the mandibular canal by dental professionals, Kainmueller et al. (2009) proposed an automated segmentation technique that combines the Dijkstra tracking algorithm with the Statistical Shape Model (SSM). This method successfully reduced the average error to 1.0mm, achieving a level of automation. Subsequently, Kim et al. (2010) presented a segmentation strategy that employs 3D panoramic volume rendering (VR) and texture analysis. Their approach captured variations in the curvature of the mandibular canal using a line tracing algorithm. Furthermore, threshold-based segmentation technologies have seen some advancements. Moris et al. (2012) employed a thresholding technique to identify the mandibular and mental foramina and then used template matching technology to recursively calculate the optimal path between them, leveraging strong prior knowledge to achieve effective segmentation results. Building on Mori et al.’s work, Onchis-Moaca et al. (2016) enhanced template matching technology by using the anisotropic generalized Hough transform of the Gabor filter, significantly improving computational efficiency. However, these methods suffer from excessive reliance on prior knowledge and limited generalizability. On the other hand, to tackle the low contrast of CBCT images, Abdolali et al. (2017) innovatively employed low-rank matrix decomposition to enhance image quality, thereby increasing the visibility of the mandibular canal in the shape model. Similarly, Wei and Wang (2021) utilized windowing and K-means clustering algorithms for data enhancement to improve the mandibular canal’s visibility and subsequently deployed two-dimensional linear tracking coupled with tetranomial fitting for segmentation. In summary, traditional segmentation methods either depend excessively on prior knowledge or require significant manual intervention, leading to pronounced human-induced biases.

### 2.2 CNN-based segmentation method

In recent years, CNN-based segmentation methods have achieved significant advancements in mandibular canal segmentation. Jaskari et al. (2020) first employed a Fully Convolutional Network (FCN) for this task, achieving a Dice coefficient of 0.57 and thus substantiating the efficacy of CNN approaches in this domain. Following this, Kwak et al. (2020) utilized thresholding technology for rapid mandibular canal localization, converting the full-volume image into a 2D slice sequence. They then employed SegNet and 3D UNet models for 2D slice-level and 3D volume-level segmentation, respectively. However, their approach did not adequately consider the structural information of the mandibular canal. To address this gap, Widiasri et al. (2022) segmented 3D images into 2D slices and utilized the Dental-Yolo algorithm for feature detection. This method computed the dimensions between the alveolar bone and the mandibular canal, allowing the model to acquire rich positional information. Additionally, to enhance segmentation accuracy and mitigate computational limitations, researchers have proposed generalized hierarchical frameworks (Lahoud et al., 2022; Verhelst et al., 2021). For instance, Verhelst et al. (2021) initially downsampled images to reduce resolution, retained only patches with foreground classes, and employed a 3D UNet in conjunction with the Marching Cubes algorithm for smoothing and segmentation. However, this method necessitates some manual input and struggles with samples that have indistinct mandibular canal boundaries. To counteract the issue of blurred boundaries, Faradhilla et al. (2021) introduced a Double Auxiliary Loss (DAL) in the loss function to make the network more attentive to the target area and its boundaries, achieving a high Dice accuracy of 0.914 on their private dataset. To combat class imbalance, Du et al. (2022) innovatively introduced a pre-processing step involving centerline extraction and region growing to identify the mandibular canal’s location. They used a fixed point as a reference to crop a localized region around the mandibular canal, thereby minimizing the impact of background samples. Despite the successes of these methods, they generally sacrifice rich global information during training, leading to a loss of structural integrity in the segmented mandibular canal.

### 2.3 Transformer-based segmentation method

In the realm of medical imaging, Transformer-based techniques have garnered considerable attention, finding applications across a range of tasks including segmentation, recognition, detection, registration, reconstruction, and enhancement (Li et al., 2023; Dosovitskiy et al., 2020). One key advantage of the Transformer architecture over Convolutional Neural Networks (CNNs) is its robust capability for global perception, allowing for a more effective understanding of global contextual information and capturing long-range dependencies. Many Transformer-based approaches have been adapted for segmentation tasks involving major human organs, and have yielded impressive results (Liu and Shen, 2022; Pan et al., 2022). For instance, Wang et al. (2021) introduced the UCTransNet model, which for the first time incorporated the Transformer into the channel dimension. By leveraging feature fusion and multi-scale channel attention, the model optimized the information integration between low- and high-dimensional spaces (Chen et al., 2021). Hatamizadeh et al. (2022a) then proposed the UNETR model, which employed the Vision Transformer (ViT) as the encoding layer. This model leveraged the Transformer’s strong global modeling capabilities to achieve excellent performance on multi-organ segmentation datasets. To address the UNETR model’s relatively weaker performance in capturing local details, Hatamizadeh et al. (2022b) introduced the Swin UNETR segmentation model. This variant ensured a global receptive field while also giving ample consideration to local details, and it has shown promising results in tasks such as brain tumor segmentation. Specifically in the context of mandibular canal segmentation, Jeoun et al. (2022) introduced the Canal-Net, a continuity-aware context network designed to help the model understand the spatial structure of the mandibular canal. This approach achieved a Dice coefficient of up to 0.87. These outcomes provide compelling evidence to suggest that the Transformer’s strong context-aware capabilities could be particularly effective for mandibular canal segmentation tasks. However, it is worth noting that research in Transformer-based mandibular canal segmentation is still in its nascent stages. Recognizing the unique challenges and characteristics of mandibular canal segmentation, we sought to improve upon the Swin UNETR model. Our modified approach has yielded promising segmentation results, underscoring the potential utility of Transformer-based architectures in this domain.

## 3 Methods

### 3.1 Data preprocessing

**FIGURE 1:**
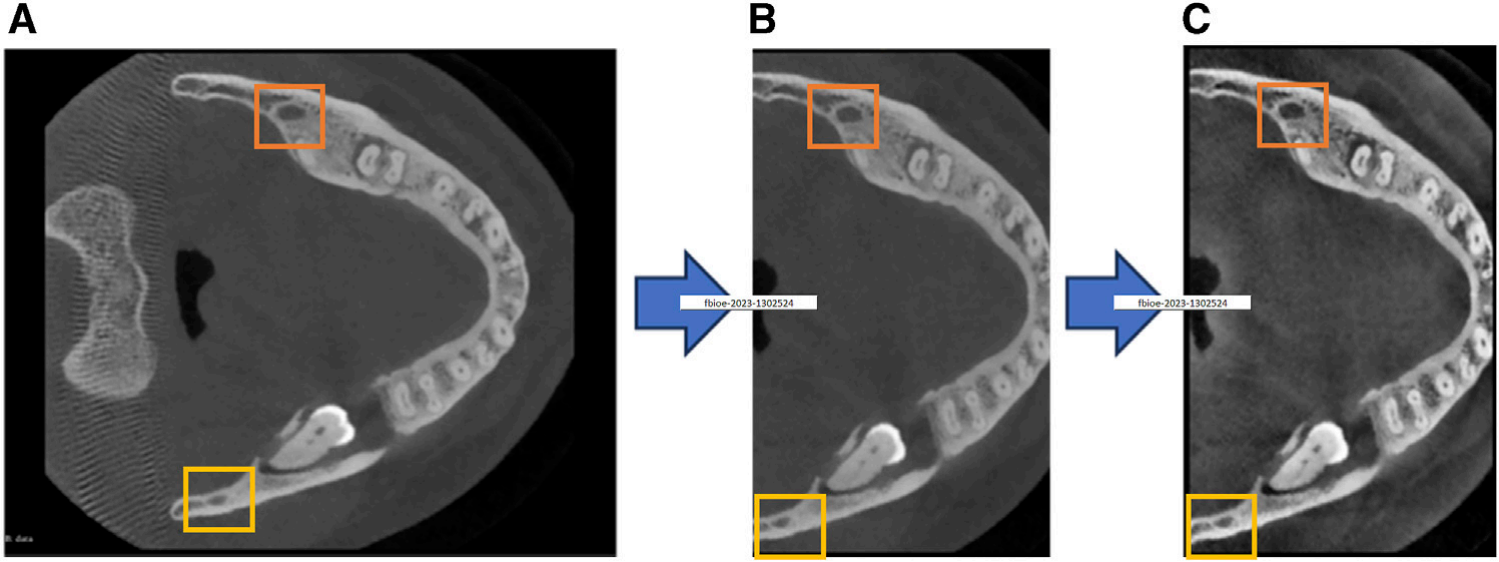
Our proposed preprocessing process, **(A)** represents the original image, **(B)** represents the cropped image, and **(C)** represents the contrast-enhanced image.

Considering the impact of preprocessing on model performance, we employ a comprehensive set of preprocessing steps to address existing challenges in CBCT imagery and thereby enhance the segmentation accuracy of the mandibular canal. The specific process is shown in Figure 1. The rectangular box in Figure 1 highlights the changes in the mandibular canal.

#### 3.1.1 Volume cropping

The proportion of voxels representing the mandibular canal in the entire CBCT image is exceedingly small, exacerbating the class imbalance between foreground and background. This imbalance adversely affects the model’s segmentation performance, as illustrated in Figure 1A. To address this challenge, we introduce a cropping technique based on the localization of the mandibular foramen. This approach aims to identify the largest connected domain of the mandibular canal by locating the jaw foramen, as depicted in Figure 1B. This method locates the positional relationship between the mandibular foramen and chin foramen in the labeled information, and then maps this positional relationship to the original image for cropping. Following this preprocessing step, the total number of voxels is reduced by approximately 60%. This reduction not only enhances the model’s convergence speed and segmentation accuracy but also minimizes hardware resource consumption.

#### 3.1.2 Contrast enhancement

In CBCT imaging, the gray values of the mandibular canal and surrounding tissues are often similar, which obscures the boundary of the mandibular canal. Further complicating the matter, some CT devices may produce images with low resolution and blurriness, making it difficult to differentiate the mandibular canal from adjacent structures. To overcome these challenges, we employ Contrast-Limited Adaptive Histogram Equalization (CLAHE) to enhance image contrast, thereby improving the model’s segmentation performance. This enhancement is demonstrated in Figure 1C.

### 3.2 Mandibular canal segmentation network structure

**FIGURE 2:**
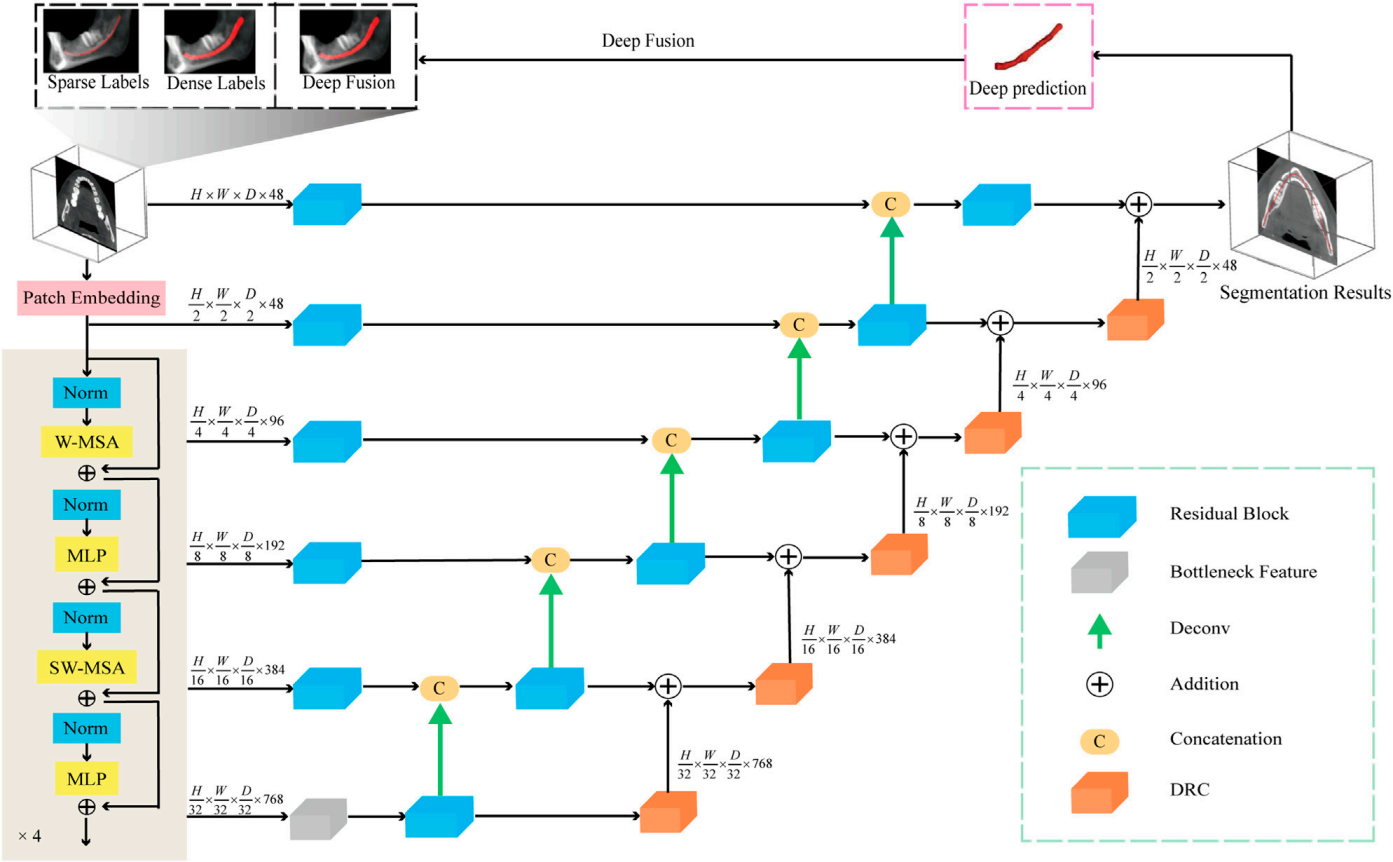
The network diagram used in this article consists of a Transformer encoding module, a decoding module, and a feature fusion module. In addition, the model accepts three types of labels: sparse label, dense label, and deep fusion label.

We employ the aforementioned preprocessing techniques on the CBCT images and use them as input for the segmentation network. In the encoder portion of the network, a 4-layer Swin Transformer serves as the feature extractor. This architecture leverages the Transformer’s robust capability for global modeling, allowing it to focus more effectively on the overall structural features of the mandibular canal, compared to traditional CNN-based feature extractors. The decoder part of the network adheres to the conventional U-Net decoding structure. In this design features extracted by the encoder are connected to the decoder via skip connections at each scale. At each stage of the encoder i the output features are reshaped to size $\frac{H}{2^{i}}\times \frac{W}{2^{i}}\times \frac{D}{2^{i}}$ , which are then fed into a residual module consisting of two 3 × 3 × 3 convolutional layers. Subsequently, the feature map is upsampled by 2 times using a deconvolution layer, and is concatenated with the output of the previous layer and fed into the residual module. Finally, the output of the residual module is sent to the DRC module to achieve pixel-level feature fusion with the previous layer features. The final segmentation result is calculated by using a 1 × 1 × 1 convolutional layer and a sigmoid activation function. It restores the spatial dimensions of the feature map through a series of five upsampling operations, as shown in Figure 2.

**FIGURE 3:**
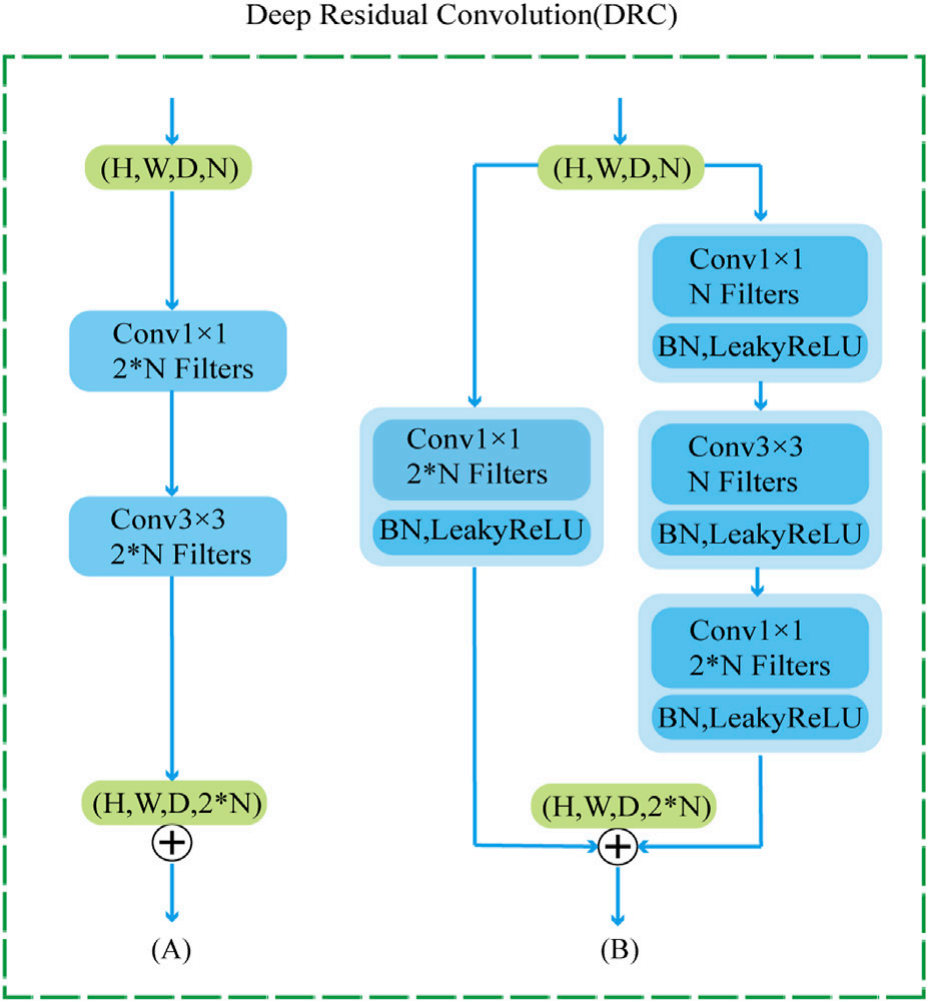
DRC module structure diagram, where **(A)** represents the traditional convolution module **(B)** represents our proposed DRC module.

To further improve the network’s ability to perceive the details of the mandibular canal, we introduce a feature fusion strategy of element-by-element addition and use the DRC (Deep Residual Convolution) module for each decoding layer to further extract features, as shown in Figure 3B. Comparing with traditional CNN structure, as shown in Figure 3A, this module is mainly composed of two branches: the first branch consists of a 1 × 1 convolution, the second branch consists of two 1 × 1 and a 3 × 3 convolution, and to improve the expressiveness of the convolution, we perform normalization and ReLU activation operations after each convolution operation. The output of the DRC module can be expressed as:

$$DRC=F{\left(X,Y\right)}_{L}+F{\left(X,Y\right)}_{R}$$ (1)

among them, X represents the input data, ${F\left(X,Y\right)}_{L}$ represents the output of the left branch, and ${F\left(X,Y\right)}_{R}$ represents the output of the right branch. The extracted features are fused layer by layer at the pixel level to obtain the fused feature map $F\left(x,y\right)$ :

$$F\left(x,y\right)=F_{n}\left(x,y\right)+DRC\left(F_{n-1}\left(x,y\right)\right)$$ (2)

where $F\left(x,y\right)$ represents the pixel position in the feature map, and *n* represents the nth decoder layer. Through this fusion strategy, the model can learn more information from different feature map.

### 3.3 Deep Label fusion

To optimally leverage our set of 256 sparsely labeled data, we introduce an innovative approach for pseudo-label generation. Initially, the model is trained using densely annotated data, after which it generates pseudo-labels for the 256 sparsely annotated samples. Compared to the original sparse labels, these pseudo-labels offer a richer semantic context but may lack adequate connectivity. To address this limitation, we implement an intelligent label fusion algorithm. This method first integrates instance level features of circular extended labels and newly generated pseudo labels through interpolation. More specifically, the pseudo-labels contribute valuable semantic insights, while the circular extension labels provide precise boundary delineation. We have coined this method “Deep Label Fusion,” and employ it to create an augmented dataset for this study. Utilizing this extended dataset for pre-training the prediction model led to a notable improvement in the Dice metric, particularly when compared to the performance achieved with the original set of 256 circularly extended labels.

### 3.4 Loss function analysis

The primary objective of the loss function in medical image segmentation tasks is to quantify the discrepancy between the predicted segmentation outcomes and the ground-truth labels. Given that the mandibular canal is a tubular structure, its connectivity is a crucial consideration. In 2021, Shit et al. introduced a loss function designed to take into account both vessel topology and connectivity, known as centerline Dice (clDice). This function is computed based on the intersection between the segmentation output and the extracted cartilage scaffold. Importantly, clDice is adept at evaluating the connectivity of tubular anatomical features. In our research, we employ clDice as the loss function for training the network. The expression for the clDice function is as follows:

$$T_{p}\left(S_{P},V_{L}\right)=\frac{\left|S_{P}\cap V_{L}\right|}{\left|S_{P}\right|},$$ (3)

$$T_{s}\left(S_{L},V_{P}\right)=\frac{\left|S_{L}\cap V_{P}\right|}{\left|S_{L}\right|},$$ (4)

$$L_{clDice}=-2\times \frac{T_{p}\left(S_{P},V_{L}\right)\times T_{s}\left(S_{L},V_{P}\right)}{T_{p}\left(S_{P},V_{L}\right)\times T_{s}\left(S_{L},V_{P}\right)},$$ (5)

among them, *V*_*L*_ and *V*_*P*_ refer to the predicted results and real labels of network segmentation, respectively, *S*_*P*_ and *S*_*L*_ refer to the soft skeleton extracted from *V*_*L*_ and *V*_*P*_, respectively, *Tp(S*_*P*_ *, V*_*L*_ *)* refers to the topological accuracy, *Ts(S*_*L*_ *, V*_*P*_ *)* is the topological sensitivity. *L*_*clDice*_ is the harmonic mean of the above two metrics to focus on object connectivity. The total loss function *L*_*total*_ combines Dice Loss and clDice Loss, the formula is as follows:

$$L_{total}=\left(1-\lambda \right)L_{Dice}+\lambda L_{clDice},$$ (6)

where *λ* is a scaling factor.

## 4 Data and implementation details

### 4.1 Data

The CBCT dataset utilized in this study was supplied by Cipriano et al. (2022b) and exists in two versions: old and new. The old dataset comprises 91 3D densely annotated primary datasets and 256 2D sparsely annotated auxiliary datasets. This primary dataset is further divided into 68 training sets, 8 validation sets, and 15 test sets. The spatial resolutions of these CBCT scans range from 148 × 272 × 334 to 171 × 423 × 462, featuring a voxel size of 0.3 × 0.3 × 0.3 mm³. Conversely, the new dataset consists of 153 densely annotated primary datasets and 290 sparsely annotated auxiliary datasets. The spatial resolution in this new version ranges from 148 × 265 × 312 to 178 × 423 × 463. Additionally, the training set in this new version has been expanded to include 130 datasets. To maintain a fair and rigorous comparison with other studies, all comparative analyses were conducted using the old dataset. Moreover, to demonstrate the cutting-edge nature of our research, we also conducted verifications using the new dataset version.

### 4.2 Experimental details

Our experiments are implemented using NVIDIA Tesla V100S in the PyTorch and MONAI deep learning libraries. During the preprocessing, we processed the raw data offline by the proposed jaw-foramen localization-based volume cropping method and image contrast enhancement method. During the training process, Diceloss and clDiceloss are used as the loss function of the model, the Adam optimizer with momentum (*μ* = 0.99) is used, the initial learning rate is set to 0.0001, and the learning rate is automatically adjusted using cosine annealing, and the batch size is set to 1, and the number of iterations of the model is uniformly set to 500. In our experiments, to reduce memory usage, we use a 96 × 96 × 96 sliding window with a stride of 48 to crop the original CBCT image, and then feed the cropped image into the network for training. After outputting the predicted patch, we restore the output predicted patch to the original image size by stitching.

### 4.3 Evaluation indicators

In the test phase, we use four commonly used evaluation indicators for segmentation tasks to evaluate the performance of the model: Dice coefficient (Dice), Intersection Over Union (IOU), Hausdorff distance (HD):

#### 4.3.1 Dice coefficient (Dice)

The Dice coefficient is a set similarity measurement function, which is usually used to calculate the similarity between two samples, and the value range is [0, 1].

$$Dice=\frac{2TP}{FP+2TP+FN}$$ (7)

#### 4.3.2 Intersection over union (IOU)

The IOU indicator calculates the overlap rate of predicted results and real results, that is, the ratio of their intersection and union.

$$IOU=\frac{A\cap B}{A\cup B}$$ (8)

#### 4.3.3 Hausdorff distance (HD)

The HD indicator is a metric used to measure the similarity or difference between two sets.

$$H\left(A,B\right)=\max\left\{h\left(A,B\right),h\left(B,A\right)\right\}$$ (9)

where TP represents true positives, TN represents true negatives, FP represents false positives, FN represents false negatives, A represents the set of true labels, and B represents the set of predicted segmentations.

## 5 Results

### 5.1 Evaluation of result

**FIGURE 4:**
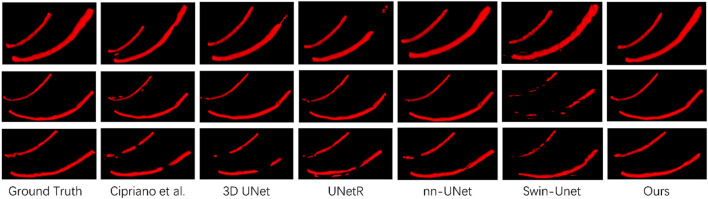
Comparison of visualization results of different segmentation methods.

To prove the effectiveness of our proposed mandibular canal segmentation method, we conducted a performance evaluation. The specific results are as follows: only trained on 91 dense data, average Dice = 0.815, average IoU = 0.69, average clDice = 0.93, the average HD95 = 5 mm, all evaluation indicators have proved the excellence of our proposed mandibular canal segmentation method. In addition, to prove the advanced nature of our proposed method, we also compared and analyzed it with existing methods, and the specific comparison results are shown in Table 1. From the comparison results, it can be seen that the improvement of the segmentation method we proposed is very significant. Compared with the most advanced method that also uses 91 densely labeled data, our Dice index has increased by 4.5%. In addition, using 256 sparse data for pre-training and our proposed deep label fusion strategy for training, the Dice index reached 0.824 and 0.840, respectively. The specific visual comparison results are shown in Figure 4.

**TABLE 1:** Comparison of results of different segmentation methods.

Test	Methods	Training set	HD	IoU	clDice^#^	Dice
1	Jaskari et al. (2020)	Cir. Exp.	—	0.39	—	0.56
2	Ours	Cir. Exp.	7.844	**0.405**	0.845	**0.573**
3	Cipriano et al., (2022a)	3D Ann.	—	0.61	—	0.75
4	Usman et al., (2022)	3D Ann.	—	0.79	—	0.77
5	3D UNet	3D Ann.	16.048	0.558	0.809	0.709
6	nn-UNet	3D Ann.	6.363	0.665	**0.935**	0.796
7	UNetR	3D Ann.	8.027	0.569	0.823	0.722
8	Swin-Unet (Cao et al.)	3D Ann.	7.072	0.482	0.733	0.640
9	Ours	3D Ann.	**5.002**	**0.692**	0.933	**0.815**

Bold represents the optimal result. # is the measurement standard for tubular structure proposed by Shit et al. (2021).

### 5.2 Ablation experiment

#### 5.2.1 Preprocessing

In Section 3.2, we proposed two data preprocessing methods, namely, the cropping method based on jaw hole positioning and the contrast enhancement method based on Contrast-Limited Adaptive Histogram Equalization. Figure 4 shows our proposed preprocessing method in detail. We have analyzed the effectiveness of the two proposed methods, and the specific results are shown in Table 2. It can be seen from the table that the Dice index has increased by 2.7% after data preprocessing.

**TABLE 2:** Quantitative analysis results of different preprocessing methods on model performance.

Input images	HD_95_ (mm)	IoU	clDice	Dice
Original	6.355	0.656	0.912	0.788
Volume Cutting	5.008	0.684	0.927	0.810
Contrast Enhancement	**5.000**	**0.692**	**0.933**	**0.815**

Bold values are reports the optimal result.

#### 5.2.2 Feature fusion

To deeply study the impact of our proposed feature fusion strategy on the performance of mandibular canal segmentation, we conduct a series of ablation experiments and summarize the experimental results in Table 3. The results show that the feature fusion strategy plays an important role in the mandibular canal segmentation task. The Dice coefficient using this feature fusion strategy is 0.788, which is significantly improved compared to the case where this module is not used. In addition, we conduct a comparative analysis of the proposed DRC module and traditional convolution. This design can effectively enhance the representation ability of the model and help to further optimize our proposed feature fusion strategy to improve segmentation performance.

**TABLE 3:** Quantitative results of different feature fusion methods.

Test	HD_95_ (mm)	IoU	clDice	Dice
Baseline	10.320	0.629	**0.92**	0.769
Baseline+C	9.550	0.642	0.894	0.777
Baseline+DRC	**6.355**	**0.656**	0.912	**0.788**

Among them, C means to use the traditional convolution module, and DRC means to use the deep residual convolution module. Bold values are reports the optimal result.

#### 5.2.3 Loss function analysis

**FIGURE 5:**
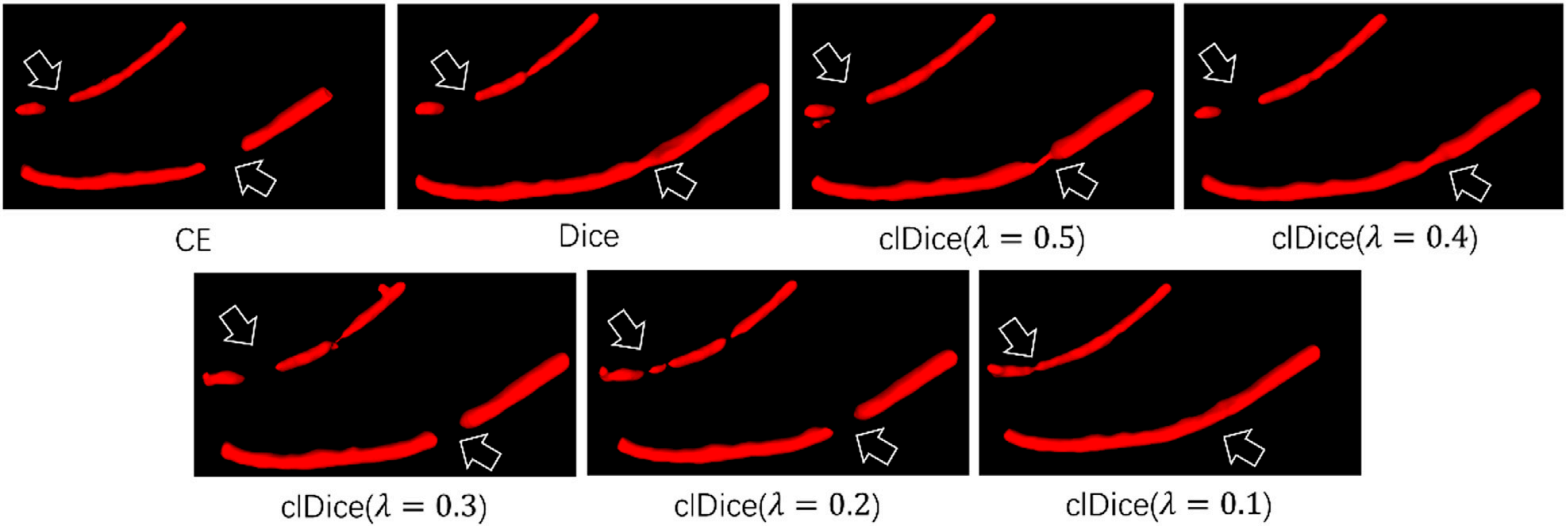
Visual segmentation results obtained by training the network with different loss functions.

In our work, we use clDice Loss as the loss function to train the network. In order to prove that clDice Loss is helpful in improving the mandibular canal segmentation effect, we compared clDice Loss with Cross-Entropy (CE) Loss and Dice Loss. The specific results are shown in Table 4. We found that using clDice Loss as the loss function achieved the optimal Dice coefficient of 0.815. In addition, we also compared the impact of different hyperparameters *λ* in clDice Loss on model segmentation performance. The specific visual comparison results are shown in Figure 5. The broken part of the mandibular canal in the segmentation result is marked with arrows. From the figure, it can be clearly seen that the segmentation result has the best connectivity when *λ* = 0.1.

**TABLE 4:** Quantitative results of training models with different loss functions.

Loss function	HD_95_ (mm)	IoU	clDice	Dice
Cross-Entropy (CE)	8.207	0.660	0.907	0.790
Dice	7.828	0.665	0.888	0.795
clDice ( λ=0.5 )	9.392	0.656	0.909	0.787
clDice ( λ=0.4 )	6.958	0.680	0.927	0.806
clDice ( λ=0.3 )	9.550	0.660	0.907	0.789
clDice ( λ=0.2 )	5.005	0.681	0.927	0.806
clDice ( λ=0.1 )	**5.002**	**0.692**	**0.933**	**0.815**

Bold values are reports the optimal result.

#### 5.2.4 Deep label fusion

In Section 3.3, we proposed a deep label fusion method, which generated 256 fused labels on a sparse dataset, forming a new deep fusion dataset. This deep fusion dataset contains richer semantic information compared to sparse datasets, which can better guide model training. To verify the effectiveness of this method, we conducted a performance evaluation, and the specific evaluation results are shown in Table 5. From the table, it can be seen that the Dice index has been significantly improved when using our proposed deep fusion label for pre training. When using 3D Ann data for training, using our method for pre training is 1.6% higher than using circular extended data for pre training Dice index.

**TABLE 5:** Analysis of training results using different labels.

Test	Pre-training set	Training set	HD	IoU	clDice	Dice
1	—	3D Ann.	5.002	0.692	0.933	0.815
2	Cir. Exp.	3D Ann.	4.061	0.704	0.947	0.824
3	Deep fusion	3D Ann.	3.213	0.727	0.960	0.840
4	Deep fusion	ToothFairy	**2.947**	**0.731**	**0.961**	**0.844**

Notes: 3D Ann indicates densely labeled data, Cir. Exp. represents circle expansion data, Deep fusion represents synthetic data sets, and ToothFairy represents new version data sets. Bold values are reports the optimal result.

## 6 Discussion

In this study, we propose a Transformer-based method for automatic segmentation of the mandibular canal, which is capable of simultaneously focusing on local fine-grained details and global semantic information of the mandibular canal to segment the mandibular canal with highly consistent accuracy across the entire CBCT image. We validate the method on the largest mandibular canal segmentation dataset, and the evaluation index Dice coefficient exceeds previous research methods.

Due to the low contrast in CBCT images and the close similarity in grayscale values between the mandibular canal and surrounding tissues, neural networks face difficulties in effectively distinguishing the boundaries of the mandibular canal (Waltrick et al., 2013). Furthermore, the mandibular canal constitutes only a minute fraction of the total CBCT image volume, leading to a pronounced class imbalance between foreground and background. This imbalance causes the network to disproportionately focus on background features (Dai et al., 2023). To address these challenges, we employ two pre-processing techniques aimed at mitigating issues related to blurred boundaries and class imbalances: a cropping method for automated localization of the mandibular and mental foramina, and Contrast-Limited Adaptive Histogram Equalization (CLAHE) for image contrast enhancement. By eliminating extraneous information and enhancing the contrast between the mandibular canal and its surrounding tissue, these techniques facilitate precise localization and segmentation of the mandibular canal. The efficacy of this contrast enhancement approach has also been successfully validated in two-dimensional microscopic images as per Wu et al. (2022). As demonstrated in Section 5, the implementation of these two preprocessing methods results in an approximate 4% improvement in Dice coefficient performance.

Secondly, to maintain the connectivity of the segmented mandibular canal, we incorporated the Transformer architecture into the segmentation task. This enables the network to learn both the local fine-grained details and the global semantic information pertinent to the mandibular canal (Liu et al., 2021). Given the small volumetric proportion of the mandibular canal in the CBCT images, we introduced a pixel-level feature fusion strategy to augment the network’s segmentation performance. The deployment of the Deep Residual Convolution (DRC) module further enriches the network’s perception of intricate details. Previous studies have substantiated the efficacy of feature fusion strategies in enhancing the segmentation performance for small and indistinct targets (Dai et al., 2023). Our empirical tests show that the feature fusion module not only improves segmentation performance but also accelerates model convergence, reducing training time by as much as 50%. This acceleration is likely attributed to the enhanced perceptual capabilities conferred by the module, partially ameliorating the slow convergence typically associated with Transformer models. Regarding the loss function, we employed the centerline Dice (clDice) loss function to better account for the tubular topology of the mandibular canal (Shit et al., 2021). As evidenced in Section 5, there was a 1% increase in the Dice coefficient, corroborating the effectiveness of this method in improving the segmentation of tubular structures. Figure 5 clearly demonstrates enhanced connectivity in the segmentation results, an outcome of clDice loss function’s calculation of connectivity disparities between segmented outcomes and the extracted cartilage scaffolding. This quantification allows the network to focus more on ensuring connectivity in the segmentation results, thereby significantly enhancing the morphological integrity of the mandibular canal’s tubular structure. Similar findings are reported in Huang et al. (2022) and Pan et al. (2021). Additionally, we leveraged sparse existing data to generate an augmented dataset through our proposed Deep Label Fusion technique. Compared to pre-training on the circle-extension dataset, our method resulted in a 1.6% increase in the Dice coefficient, reaching a score of 0.840. When validated on the new version of the ToothFairy dataset, the Dice coefficient was further improved to 0.844.

In the segmentation performance of CBCT images, our method achieved the highest performance on the public mandibular canal dataset (Cipriano et al., 2022b). Although this research work achieved the best segmentation results overall, there are still certain limitations. First of all, compared with the CNN network, the convergence speed of this network needs to be improved. Secondly, because the pixel changes of the mandibular canal at the mandibular and mental foramen are not obvious, the segmentation effect of the mandibular canal at the head and tail of the foramen is poor. Therefore, we will focus on the first and last features in future research to further improve the accuracy of the model.

## 7 Conclusion

In this study, we introduce a Transformer-based method for the robust segmentation of the mandibular canal. Our approach adeptly addresses key challenges, including morphological preservation of the mandibular canal, class imbalance, and ambiguous boundaries, subsequently achieving substantial improvements in segmentation metrics. Firstly, we employ Contrast-Limited Adaptive Histogram Equalization (CLAHE) to enhance image contrast, substantially ameliorating the low-contrast issues inherent to original CBCT scans. This step results in a notable increase in the model’s segmentation accuracy. Secondly, we implement an image cropping strategy founded on mandibular foramen localization. This alleviates the class imbalance issue and substantially reduces extraneous background information, streamlining the segmentation process. Further, we introduce a specialized pixel-level feature fusion module known as the Deep Residual Convolution (DRC). This module not only amplifies the model’s sensitivity to fine details in smaller targets such as the mandibular canal but also accelerates the convergence speed of the model, partially mitigating the known slow-convergence issue associated with Transformer architectures. To improve the topological integrity of the segmented mandibular canal, we utilize the centerline Dice (clDice) loss function. This forces the network to concentrate on maintaining the connectivity of the segmented structures, enhancing the morphological integrity of the mandibular canal. Lastly, we deploy a Deep Label Fusion technique to mine further information from the original, sparsely-annotated dataset. This step significantly bolsters the model’s segmentation performance. Our method was rigorously evaluated on a publicly available mandibular canal dataset. The empirical results demonstrate that our proposed segmentation approach outperforms existing methods, underscoring its strong potential for application in the domain of mandibular canal segmentation.

## Data Availability

The original contributions presented in the study are included in the article/supplementary material, further inquiries can be directed to the corresponding author.
